# STAFFS’ PSYCHOSOCIAL WORK ENVIRONMENT IN RELATION TO RECIPIENT SATISFACTION IN HOME CARE SERVICES

**DOI:** 10.1093/geroni/igac059.2568

**Published:** 2022-12-20

**Authors:** Ingemar Kåreholt, Dan Lundgren, Zarina Nasar Kabir, Anne-Marie Boström

**Affiliations:** Jönköping University, Jönköping, Jonkopings Lan, Sweden; Municipality of Värnamo, Värnamo, Jonkopings Lan, Sweden; Karolinska Institutet, Huddinge, Stockholms Lan, Sweden; Karolinska Institutet, Huddinge, Stockholms Lan, Sweden

## Abstract

In accordance with ‘aging in place’ policy, older persons in Sweden are increasingly encouraged to continue living at home and if necessary be supported by home care service (HCS). Studies have examined whether the work environment of staff has an impact on the experiences and the wellbeing of the older persons in nursing homes, but few have examined such associations in HCS. The setting was 16 HCS work units. Two surveys were sent, one to staff on psychosocial working conditions, one to care recipients on care satisfaction. For each work unit, data on individual recipient satisfaction was matched to average values on psychosocial work conditions. Outcomes analyzed with linear regressions were overall recipient satisfaction, based on one question, and indexes on: assessment of implementation of services, contact with staff, and sense of security. Index on treatment by staff was analyzed with ordered logistic regressions due to skewed distribution. We used cluster correlated standard errors (clustering on work units). Results showed that good working conditions are important for recipient satisfaction, specifically overall recipient satisfaction, treatment by staff, and sense of security. Psychosocial work factors most important were work group climate, overall job strain, sense of mastery, job control, frustrated empathy, balancing competing needs, balancing emotional involvement, and lack of recognition. Having more home help hours was associated to stronger relation between working conditions and recipient satisfaction, especially with overall recipient satisfaction and treatment by staff as outcomes.

